# Reactivation of Smac-mediated apoptosis in chronic lymphocytic leukemia cells: mechanistic studies of Smac mimetic

**DOI:** 10.18632/oncotarget.8462

**Published:** 2016-03-29

**Authors:** Kumudha Balakrishnan, Min Fu, Francesco Onida, William G. Wierda, Michael J. Keating, Varsha Gandhi

**Affiliations:** ^1^ Department of Experimental Therapeutics, The University of Texas MD Anderson Cancer Center, Houston, Texas, USA; ^2^ Department of Hematology Unit, Fondazione IRCCS Ospedale Maggiore Policlinico, University of Milan, Milan, Italy; ^3^ Department of Leukemia, The University of Texas MD Anderson Cancer Center, Houston, Texas, USA

**Keywords:** CLL, Smac mimetic, IAPs, apoptosis, XIAP

## Abstract

Dysfunctional apoptotic machinery is a hallmark feature of chronic lymphocytic leukemia (CLL). Accordingly, targeting apoptosis regulators has been proven a rational approach for CLL treatment. We show that CLL lymphocytes express high levels of XIAP, cIAP1, and cIAP2 compared to normal lymphocytes. Smac mimetic, Smac066, designed to bind to BIR3-domain of IAPs, induce apoptosis in primary CLL cells (*n*=71; *p*<0.0001), irrespective of prognostic markers. Apoptosis was mediated by diminished levels of IAPs (XIAP-*p*=0.02; cIAP-*p*<0.0001) and increased activation of caspases-8, −9, −3. The caspase-cleavage was in direct association with the levels of apoptosis (r^2^=0.8 for caspases-8, −9, −3). Correlative analysis revealed a direct relationship between reduction in IAPs and degree of apoptosis (r^2^=0.6 (XIAP); 0.5 (cIAP2)). There was a strong association between apoptosis, IAP-degradation, and concurrent caspase-activation. Pan-caspase inhibitor Z-Vad-fmk reversed the degradation of Mcl-1, but not IAPs suggesting that smac066 is selective to IAPs, however, Mcl-1 degradation is through caspase-mediated cleavage. Immunoprecipitation experiments revealed physical interaction between caspase-3 and XIAP that was disrupted by smac066. Importantly, XIAP and cIAP2 were markedly induced in bone-marrow and lymph-node microenvironments, providing a basis for IAP antagonists as anti-tumor agents in CLL. Smac066 synergized with ABT-737, revealing a mechanistic rationale to jointly target BH3 and BIR3 domains.

## INTRODUCTION

Chronic lymphocytic leukemia (CLL) is the most common form of adult leukemia in the Western world. The fundamental hallmark feature of CLL biology is defective apoptosis, a major target for activation. Resistance to apoptosis in CLL stems primarily from high levels of Bcl-2-family anti-apoptotic proteins [1] and inhibitor-of-apoptosis proteins (IAPs) [2, 3], [4]. IAPs inhibit apoptosis by neutralizing the activities of caspases, a family of proteases that act in concert to execute apoptosis or programmed cell death [4, 5]. Eight IAPs have been identified. The protein structures of IAPs contain one or more baculovirus IAP repeat (BIR) domains, which are essential for the interaction of IAPs with caspases. The carboxyl end consists of a RING zinc finger domain with E3 ubiquitin ligase activity, which facilitates auto-degradation of IAPs. IAPs, such as cellular IAP1 (cIAP1) and cIAP2, also contain a caspase-associated recruitment domain (CARD) between the BIR3 domain and the C-terminal RING domain [6]. Previous studies reported that while the BIR2 domain or the linker between BIR1 and BIR2 in X-linked IAP (XIAP) interacts with the tetrapeptide binding motif of cellular caspases-3 and −7, the BIR3 domain primarily targets the pro-apoptotic activity of caspase-9 [7, 8].

The endogenous apoptosis-inducing factor Smac (second mitochondria-derived activator of caspases; also known as direct inhibitor of apoptosis protein-binding protein with low pI (DIABLO)) is a mitochondrial protein that is released into the cytosol in response to apoptotic stimuli [9]. Smac functions as an endogenous antagonist of IAP family proteins, XIAP, cIAP1, and cIAP2 that inhibit apoptosis by sequestering caspases. Mechanistically, the wild-type Smac protein (residues 1–184) forms a dimer in solution and interacts with both BIR2 and BIR3 domains of XIAP and or the linker region of XIAP. Such a dynamic and competitive protein-protein interaction between Smac and XIAP cooperatively neutralizes the inhibition of caspases [10].

The therapeutic strategy of restoring or reactivating apoptosis was initially investigated using a synthetic Smac peptide that mimic the tetrapeptide N-terminal Smac sequence (AVPI; Ala-Val-Pro-Ile) with higher binding affinity to IAPs. Crystal structure studies indicated that the IAP-binding tetrapeptide motif of Smac and that of caspase-9 (Ala-Thr-Pro-Phe) bind to the same conserved surface groove in the BIR3 domain of XIAP [11]. This competitive binding of Smac to XIAP leads to pro-apoptotic mechanism, whereas binding of caspases to XIAP results in pro-survival contrivance, providing a mechanistic rationale for developing Smac mimetics as anti-cancer agents for the concurrent reversal of IAP-mediated inhibition of caspases [12–14].

Several strategies have been put forth for targeting IAPs; some involve short Smac-based peptides, small-molecule mimetics, and/or antisense IAP oligonucleotides [15–17]. A Smac-based peptide containing the AVPI sequence was shown to bind to BIR3 and BIR2 domains of recombinant XIAP with kd values of 0.4–0.7 μM and 6-9 μM, respectively [18]. However, because Smac-based peptides are not cell permeable, non-peptide small-molecule mimetics are synthesized as an alternative. Monovalent small-molecule mimetics that could target either the XIAP BIR3 domain to inhibit the binding of XIAP to caspase-9 [19] or the BIR2 domain, to block the interaction of XIAP with caspase-3/−7 [20] are designed. More advanced bivalent small molecules that can bind to both the BIR2 and the BIR3 domains are also synthesized. These bivalent molecules are particularly efficient and more potent antagonists than the corresponding monovalent Smac mimetics [20–22]. Therefore, concurrent targeting of the BIR2 and BIR3 domains in XIAP is a highly attractive strategy to antagonize IAPs and promote apoptosis in cancer cells. However, bivalent mimetics have a higher molecular weight and may require parenteral administration [23].

In this study, we investigated reactivation of apoptosis in CLL with smac066, a small-molecule Smac mimetic synthesized at the University of Milan - Center for Biomolecular Interdisciplinary Studies and Industrial Applications (CISI Scrl). This monomer was shown to have high affinity for the BIR3 domain of XIAP, cIAP1, and cIAP2 (IC_50_, 110 nM), [24–26] lower-nanomolar potency in MDA-MB231 cells, and low-micromolar potency in HL-60 and PC-3 cells [24]. The Smac mimetics investigated thus far have shown cytotoxicity only in combination with other chemotherapeutic drugs [27, 28] by sensitizing a broad range of tumor cells to tumor necrosis factor alpha (TNFα) and other death ligands particularly to TNF–related apoptosis–inducing ligand (TRAIL) [21]. Smac066 exhibited pro-apoptotic activity and induced apoptosis as a single agent in CLL. Apoptosis was mediated by diminished levels of IAPs and an increase in activation of caspases 8, 9, and 3. Immunoprecipitation experiments revealed that caspase-3 and XIAP were in physical association with each other and smac066 was able to disrupt the interaction and promote caspase-3 activation. Importantly, XIAP and cIAP2 were markedly induced in bone marrow and lymph node microenvironments; promoting a paradigm shift towards anti-apoptotic mechanisms. Finally, smac066 when combined with ABT-737 exhibited synergism implicating that targeting Bcl-2 family and IAP family proteins in parallel, could be a novel strategy to promote apoptosis in CLL.

## RESULTS

### IAPs are expressed at high levels in CLL lymphocytes

**Figure 1:**
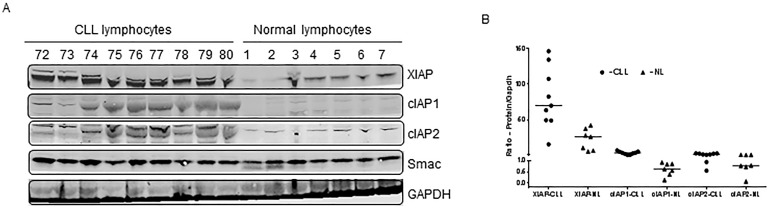
Comparison of expression levels of IAP proteins between CLL and normal lymphocytes **A.** Lymphocytes from patients with CLL (*n* = 9) and PBMCs from healthy donors (*n* = 7) were pelleted, lysed and evaluated for XIAP, cIAP1, and cIAP2 by immunoblotting analysis. GAPDH was used as a loading control. **B.** The immunoblots were quantitated and normalized to GAPDH. The horizontal lines denote median values. NL = normal lymphocytes.

The endogenous levels of target proteins (IAPs) were compared between CLL lymphocytes and normal PBMCs. Lymphocytes obtained from CLL patients (first 9 lanes; Figure 1A) and healthy donors (last 7 lanes; Figure 1A) were lysed under identical experimental conditions and evaluated for IAPs. XIAP, cIAP1, and cIAP2 proteins were higher in CLL lymphocytes than in normal lymphocytes (p=0.005, 0.0009, and 0.01, respectively; Figure 1B). Given that Smac is an endogenous pro-apoptotic protein, one would expect it to be higher in normal PBMCs; however, no difference in expression was observed between two cohorts of samples.

### Single agent Smac066, sensitizes CLL lymphocytes to apoptosis

**Figure 2:**
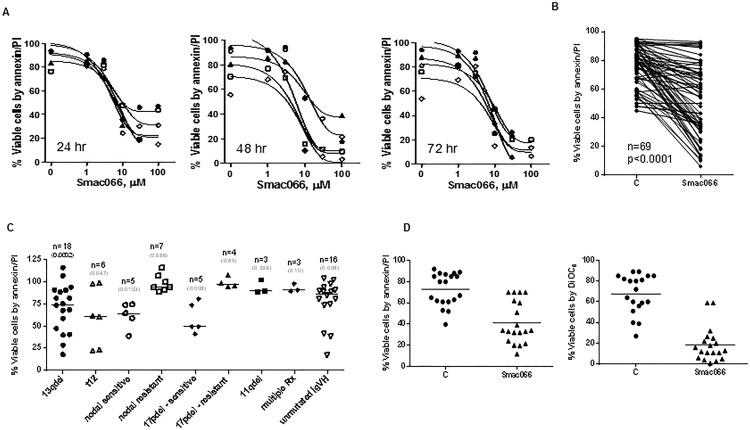
Restoration of smac066-mediated apoptosis in CLL primary cells **A.** CLL lymphocytes were incubated with serial concentrations of smac066 (0-100 μM; *n* = 5), and apoptosis was determined at 24, 48, and 72 hr by annexin V/PI binding assay. Fifty-percent inhibitory concentrations for all time points were determined using GraphPad software. **B.** Additional samples were evaluated for cell death in a similar fashion (5 μM; 24 hr; *n* = 69). The given p value is derived from paired *t*-test analyzed using Graph Pad Prizm. C = untreated CLL. **C.** Cell viability in relation to prognostic markers for individual patient samples. The values are relative to untreated time matched control (100%). The given p-values are derived from paired *t*-test between treated and untreated sample, analyzed using Graph Pad Prizm. Horizontal lines represent median values. 13q14del = miR15 and −16a locus; t12 = samples with trisomy 12 (three copies of chromosome 12); 17pdel = samples with deletion of chromosome 17p, the locus of p53, a tumor suppressor gene; 11qdel = samples with deletion of chromosome 11q, which is the locus of the ATM gene; Rx = treatments; IgVH = immunoglobulin heavy chain. **D.** Comparison of smac066-mediated apoptosis in CLL primary cells between annexin V/PI binding assay (left panel) and DiOC_6_ staining (right panel). The horizontal lines denote mean ± SEM values.

Smac066 induced apoptosis in CLL cells with median IC_50_ concentrations of 8 μM (range 6-10 μM) at 24 hr, 6 μM (range 3-15 μM) at 48 hr, and 6 μM (range 4-9 μM) at 72 hr (Figure 2A; n=5). The heterogeneity in response to apoptosis (Figure 2B; n=69; p=0.0001) was analyzed in relation to CLL prognostic markers (Figure 2C). Samples with 13q14 deletion were consistently sensitive to smac066 (n=18; p=0.0002). Samples with trisomy 12 were equally sensitive to smac066 (n=6; p=0.047). Of the samples with nodal sites and spleen, five samples were sensitive to apoptosis (p=0.01) but seven were resistant (p=0.586). Interestingly, two subsets of 17p deletion samples, one, sensitive (n=5; p=0.008) and other resistant to apoptosis (n=4; p=0.65) were observed. Samples with 11q deletion and multiple prior treatments were invariably resistant to smac066; however, the sample size was low (n=3; Figure 2C). DiOC6 staining, an alternative method of measuring apoptosis that is more specific to outer mitochondrial membrane permeabilization, was performed in parallel for comparison. In general, the results obtained with annexin V/PI binding are in linear correlation with DiOC6 staining [29]. However, in this case, apoptosis measured by DiOC6 staining was significantly higher than that measured by the annexin V/PI binding, particularly at the earlier time points (5-12 hr; Figure 2D).

### Down-regulation of IAPs with Smac066

**Figure 3:**
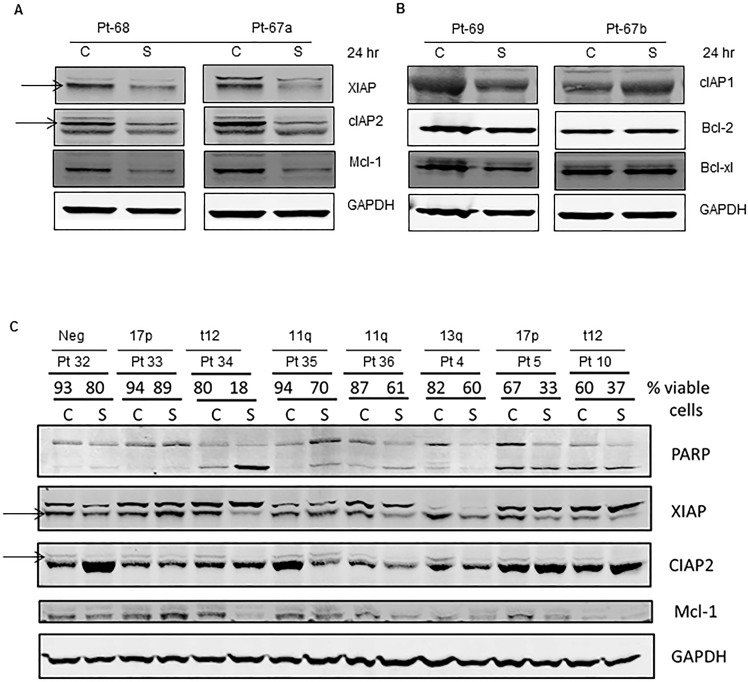

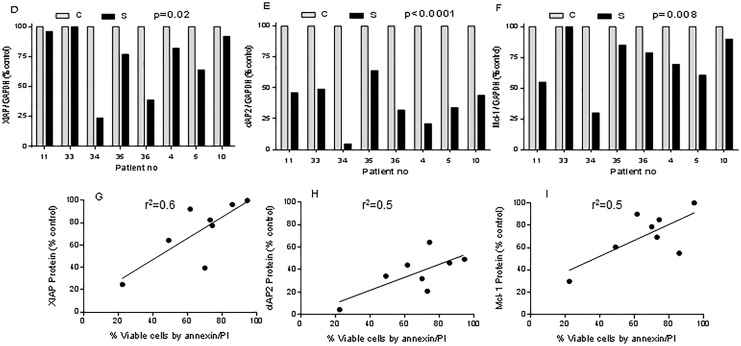

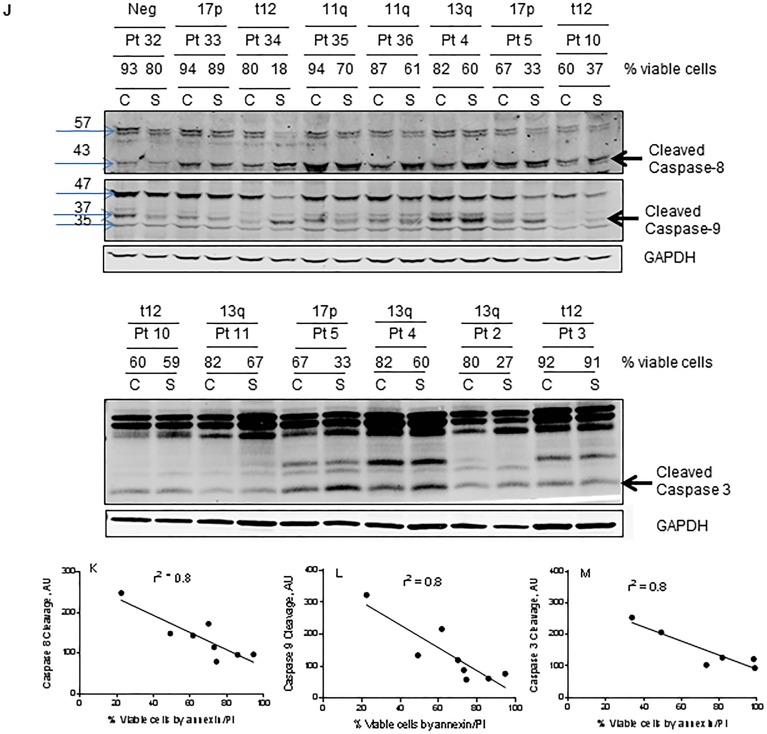
Degradation of IAPs and activation of caspases (8, 9, and 3) following smac066 treatment **A.-B.** Primary CLL cells were untreated or treated with smac066 (S) (5 μM). **A.** IAPs (XIAP, cIAP1 and anti-apoptotic Mcl-1) **B.** cIAP1 and anti-apoptotic family Bcl-2, and Bcl-xL were evaluated by immunoblotting analysis. Pt = patient; C = untreated CLL; S = smac066. **C.** Additional samples were evaluated in a similar fashion (*n* = 8). The specific bands for XIAP and cIAP2 are indicated by arrows. Prognosis and percent viable cells determined by annexin V/PI binding assay for each sample is provided. The blots originated from the same gel (the membrane is either cut into different pieces according to kD of the protein or probed with two antibodies (XIAP and cIAP2) of different species (rabbit/mouse) at the same time; this is technically feasible with LI-COR Odyssey infrared imager. **D.**-**F.** Quantitation of immunoblots (3C) for XIAP, cIAP2, and Mcl-1, normalized to GAPDH levels. **G.**-**I.** Correlation between percent viable cells and protein levels of XIAP, cIAP2, and Mcl-1 following 24-hr treatment with smac066 (5 μM; *n* = 8). The r^2^ on the correlation is obtained through linear regression analysis. **J.** Primary CLL cells were untreated or treated with smac066 (S) (5 μM) for 24 hr and caspase cleavages were measured by immunoblotting analysis. GAPDH was used as a loading control. Prognosis and percent viable cells determined by annexin V/PI binding assay for each sample is provided. The ratio between the protein of interest and its respective GAPDH is set as 100%. C = untreated CLL. **K.**-**M.** Correlation between percent apoptosis and caspase cleavage (caspases 8, 9 and 3) following smac066 treatment (5 μM; 24 hr). The r^2^ on the correlation is obtained through linear regression analysis.

Given that IAPs are potential targets of smac066, we next investigated the downstream consequences of inhibition. With 5 μM smac066, there was a significant diminution in XIAP and cIAP2 levels (Figure 3A; 24 hr). When the same samples were tested for anti-apoptotic proteins Mcl-1 (Figure 3A; 24 hr), additional samples for Bcl-2, and Bcl-xL (Figure 3B), a reduction was observed for Mcl-1 but not for Bcl-2 or Bcl-xL. Additional samples analyzed (Figure 3C; n=8) demonstrated heterogeneity for degradation of IAPs and Mcl-1 (degradation correlated with sensitivity to smac066). Overall, there was a significant reduction in protein levels following smac066 treatment (p=0.02 for XIAP, p<0.0001 for cIAP, p=0.008 for Mcl-1) (Figure 3D-3F). Correlative analysis revealed a linear relationship between reduction in protein levels and degree of apoptosis, suggesting that reactivation of apoptosis by smac066 is associated with diminution in IAP levels (Figure 3G-3I; r^2^=0.6 for XIAP, r^2^ = 0.5 for cIAP, r^2^=0.5 for Mcl-1; n=8).

### Activation of caspases with Smac066

Smac is an endogenous apoptosis-inducing factor, and its function is to neutralize the anti-apoptotic properties of IAPs, which sequester the pro-apoptotic caspases and impede their activation. On the basis of this concept, we investigated smac066-mediated apoptosis in relation to activation of caspase cascade. The lysates from same samples were evaluated for caspase activation (Figure 3J; n=8 for caspase-8 and −9 and n=6 for caspase-3). Compared to untreated samples, smac066-treated samples demonstrated significantly greater cleavage of caspase-8 (cleaved fragment of 43 KD), caspase-9 (leading to cleaved fragments of 37 and 35 KD), and caspase-3 (Figure 3J). Importantly, the levels of cleaved fragments were in direct association with the levels of apoptosis in primary CLL cells, suggesting that smac066-induced apoptosis is a direct outcome of conversion of pro-caspases into active caspases (Figure 3K-3M; r^2^= 0.8 for caspase-8 (n=8), caspase-9 (n=8), and caspase-3 (n=6)).

### Pan-caspase inhibitor, Z-Vad-fmk abrogates the smac066 mediated apoptosis

**Figure 4:**
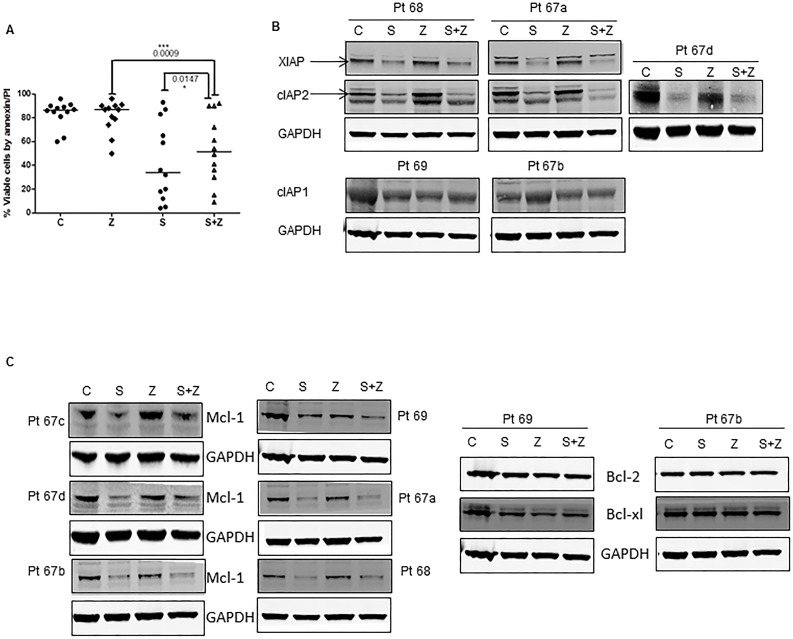

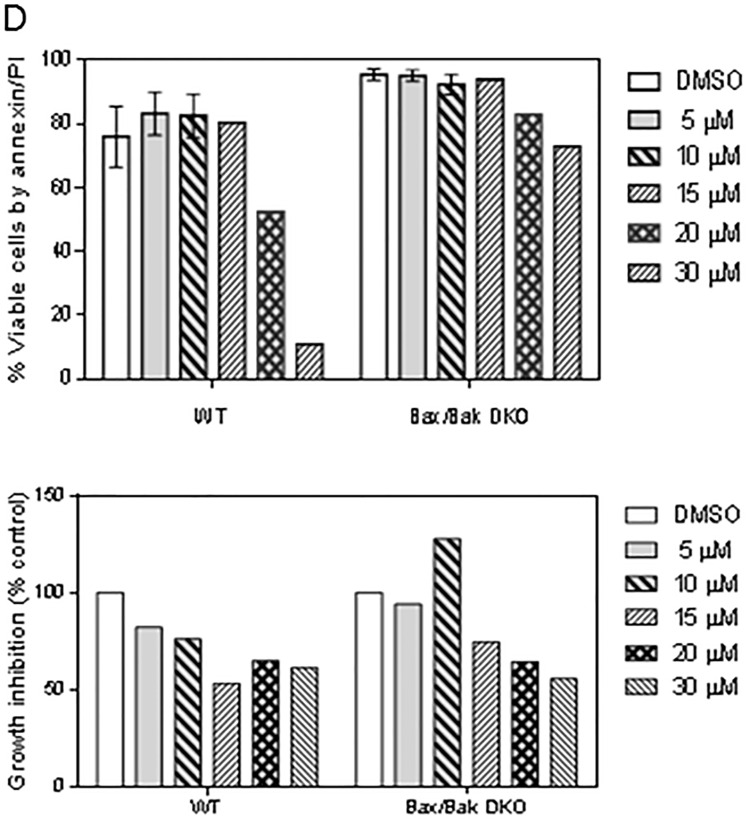
Effect of pan-caspase inhibitor Z-VAD-fmk on smac066-induced apoptosis **A.** CLL primary cells were incubated with smac066 (S) in the presence or absence of Z-VAD-fmk (Z), and percent viable cells was determined by annexin/PI binding assay (*n* = 12). The given p values are derived from paired *t*-test analyzed using Graph Pad Prizm. The horizontal lines denote median values. C = untreated CLL. **B.**-**C.** Protein levels of IAPs (XIAP, cIAP1, and cIAP2) and Bcl-2 family anti-apoptotic proteins (Bcl-2, Mcl-1, and Bcl-xL) were determined by immunoblotting. Pt 67a-d denotes that this patient arrived twice to the clinic and the immunoblot analyses were done twice. GAPDH was used as a loading control. **D.** Mouse embryo fibroblasts for wild-type (WT) or double-knockout (DKO) Bax/Bak were incubated without or with various concentrations of smac066 for 24 hr (*n* = 3). Percent viable cells were determined by annexin/PI binding assay (*n* = 3; error bars denote mean ± SEM), and percent growth inhibition was determined by the cell counting method (*n* = 3; one representative data is produced). The data are expressed as percent relative to control (untreated cells). DMSO = dimethyl sulfoxide.

To further understand the mechanism of caspase activation in smac066-induced cell death, we tested if inhibition of caspases with pan-caspase inhibitor Z-Vad-fmk would abrogate smac066-mediated apoptosis in CLL cells. Incubation with Z-Vad-fmk partially but significantly inhibited smac066-mediated apoptosis in CLL primary cells (Figure 4A; p=0.015; n=12). To further explore the mechanisms of IAP degradation and their association with caspase-mediated cleavage, protein levels of IAPs (Figure 4B) and Bcl-2 family proteins (Figure 4C) were evaluated following smac066 treatment in the presence or absence of Z-Vad-fmk. Degradation of Mcl-1 was partially reversed by the addition of Z-Vad-Fmk, however, degradation of IAPs was not restored; suggesting that while IAPs are down-regulated through degradation mechanism, Mcl-1 diminution is driven through caspase-mediated cleavage (Figure 4B and 4C).

Although Smac protein acts downstream of mitochondria, it is possible that a positive feedback loop mechanism ruptures the mitochondrial outer membrane potential and induces cytochrome c release and apoptosis, with subsequent involvement of Bax/Bak. To test this possibility, exponentially growing Bax/Bak wild-type or double-knockout mouse embryo fibroblasts were incubated with equivalent concentrations of smac066, and apoptosis was measured after 24 hr. The results showed that Bax/Bak are required for smac066-induced apoptosis, but not for growth inhibition (Figure 4D).

### Smac066 dissociates the protein-protein interaction between IAPs and caspases

**Figure 5:**
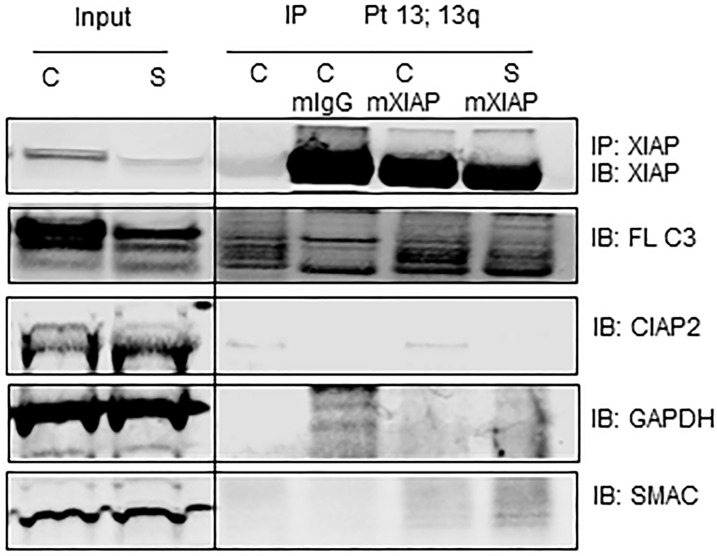
Caspase-3 associates with XIAP and smac066 disrupts the protein-protein interaction CLL primary cells were incubated without or with (5 μM; 24 hr) smac066 (S). First two lanes are inputs containing 10% whole cell lysate. Cell extracts of untreated (C) and smac066 (S) treated were immune-precipitated (IP) for none (lane 3), mouse IgG (lane 4), mouse XIAP (lane 5) and mouse XIAP (lane 6) and immuno-blotted (IB) for XIAP, full-length caspase 3 (FL- C3), cIAP2, Smac, and GADPH. Immunoprecipitating XIAP also detected full length caspase-3 demonstrating the physical association between these two molecules. Treatment with smac066 (S) compared to untreated CLL (C) diminished the XIAP protein that is associated with caspase 3 (lane 6). Other proteins such as cIAP2 and GAPDH that were present in input were not detected in IP samples.

Mechanistic studies revealed that the BIR2 and BIR3 domains of IAPs sequester caspases and neutralize their pro-apoptotic function[30]. To investigate whether the apoptosis induced by smac066 in CLL primary cells is mediated by disruption of protein–protein interaction between XIAP and caspase-3, immuno-precipitation analyses were conducted (Figure 5). The first two lanes show results for whole-cell lysates (input) probed with respective antibodies. The next four lanes show results for lysates immunoprecipitated with none (first lane) IgG mouse (second lane), mouse XIAP (third lane) and mouse XIAP (fourth lane) antibody. “C” represents untreated control and “S” represents smac066 treated. Western blot for XIAP, cIAP2, and caspase-3 confirmed that the pull-down with XIAP antibody was efficient, as indicated by the presence of XIAP, but not cIAP2. The same blots probed for caspase-3 antibody demonstrated association of caspase-3 with XIAP. Treatment with smac066 resulted in comparatively lower levels of XIAP (in input, as well as IP samples) and decrease in caspase 3 that is bound to XIAP (compare lane 5 and 6) and an increase in Smac levels (compare lanes 5 and 6). These data suggest that XIAP and caspase-3 are in physical association with each other and smac066 dissociates this protein – protein interaction in CLL lymphocytes, and displaces caspases for activation (Figure 5).

### Induction of IAPs in bone marrow or lymph node microenvironment

**Figure 6:**
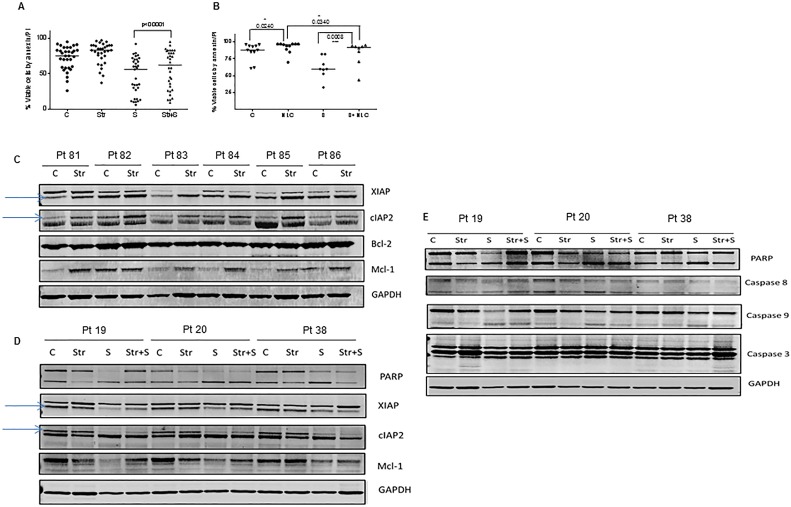
Effect of bone marrow and lymph node microenvironments on smac066-driven apoptosis CLL lymphocytes were co-cultured with NKTert stromal cells (Str) (**A.**; *n* = 33) or nurse-like cells (NLC) (**B.**; *n* = 11) in the presence or absence of smac066 (S), (24 hr) and percent viable cells was determined by annexin/PI binding assay. The horizontal lines denote median values. The given p values are derived from paired *t*-test analyzed using Graph Pad Prizm. The horizontal lines denote median values. C = untreated CLL. **C.** Primary CLL cells were co-cultured with NKtert stromal cells, and the samples were evaluated for IAPs (XIAP and cIAP2) and anti-apoptotic proteins Bcl-2 and Mcl-1. **D.**-**E.** CLL cells were incubated with NKtert stromal cells in the presence or absence of smac066, and expression of IAPs (XIAP and cIAP2), anti-apoptotic protein Mcl-1, and apoptosis marker poly(ADP-ribose) polymerase as well as caspase cleavages (caspases 8, 9, and 3) were evaluated by immunoblotting (*n* = 3). GAPDH was used as a loading control.

A growing body of evidence suggests that the resistance of CLL cells to apoptosis is mediated partly by interactions between leukemia cells and adjacent stromal cells residing in the lymphatic tissue or bone marrow microenvironment. In order to test if smac066 can overcome stromal-cell-mediated resistance to apoptosis, primary CLL cells were co-cultured with NKTert stromal cells that mimic the bone marrow stromal microenvironment, in the presence or absence of smac066. Smac066-induced apoptosis was partly but significantly abrogated in the presence of stroma (Figure 6A; n=33; p<0.0001). Similar results were obtained with nurse-like cells, which mimic the lymph node microenvironment (Figure 6B; n=11, p=0.0008). In order to understand the mechanism involved in stromal-cell-mediated resistance to apoptosis, IAPs were measured in CLL cells co-cultured with stromal cells. XIAP and cIAP2 levels were significantly induced in cells co-cultured with stromal cells than in control cells (Figure 6C; n=6). Caspase-3 cleavage was also diminished in CLL cells co-cultured with stromal cells [31]. Addition of smac066 did not abrogate the induction of IAPs (Figure 6D; n=3) or inhibition of caspase activation (Figure 6E; n=3). This finding is consistent with the report that the resistance of CLL cells to Smac mimetics is due to recurrence of cIAP2 in the lymph node microenvironment, which inhibits formation of the ripoptosome complex and apoptosis [32].

### Smac066 in combination with ABT-737 enhances apoptosis

**Figure 7:**
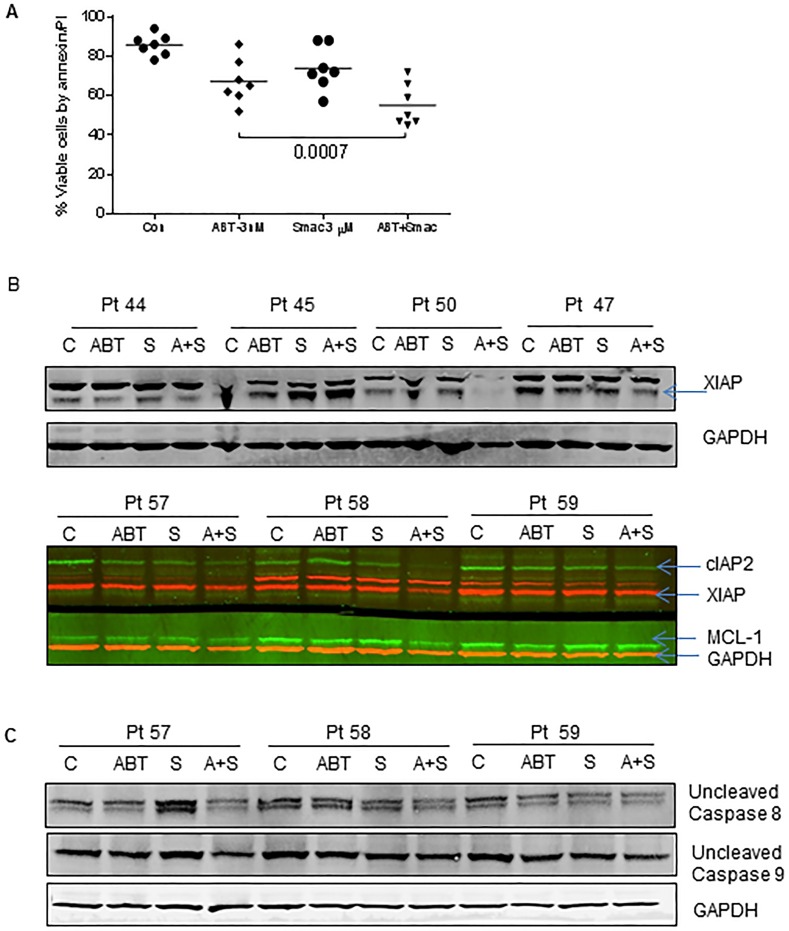
Smac066 synergize with ABT-737 **A.** Primary cells were incubated with smac066 in combination with ABT-737 and the apoptosis was measured by annexin/PI binding assay (*n* = 7). The given p values are derived from paired *t*-test analyzed using Graph Pad Prizm. The horizontal lines denote mean values. **B.** CLL primary cells were incubated with smac066 in combination with ABT-737 and IAPs and Mcl-1 were evaluated by immunoblotting analysis (*n* = 7). Red -XIAP; GAPDH and Green - cIAP2 and Mcl-1. **C.** CLL primary cells were incubated with smac066 in combination with ABT-737 and uncleaved fragment of caspases-8 and 9 were evaluated by immunoblotting analysis (*n* = 3). GAPDH was used as a loading control.

Smac066 is not a chemotherapeutic agent, but a sensitizer of apoptosis. Based on this notion, we hypothesized that smac066 should synergize with agents that prime the malignant cells to apoptosis. Because IAP family proteins as well as anti-apoptotic proteins are primarily responsible for resistance mechanisms in CLL, inhibiting both these family proteins jointly should exhibit synergy and enhance apoptosis. Based on this rationale, non-cytotoxic concentrations of smac066 (3 μM) and ABT-737 (3 nM) were added to CLL cells (Figure 7A; n=7). In comparison to apoptosis exhibited by single agents, the combination enhanced apoptosis (p=0.0007). To further explore the mechanism involved in the synergistic actions, both IAP family and Bcl-2 family anti-apoptotic protein were evaluated in these samples. XIAP and cIAP2 protein levels in the combination samples demonstrated a significant reduction compared to single agents per se suggesting that there might be an association between these two family proteins (n=7; lane 4 for each patient sample) and their inhibition could cause double insult on these cells. Interestingly, Mcl-1 levels (lane 4) and Bcl-XL (data not shown) also went down in the combination suggesting that there must be a relevant cross talk in the regulatory mechanisms of these two families of pro-survival proteins (Figure 7B). Additional experiments for caspase activation demonstrated significant decrease in the uncleaved fragments of caspases-8 and 9, in comparison to single agent smac066 or ABT-737 (Figure 7C).

## DISCUSSION

CLL is characterized by defective apoptosis, and every effort to eradicate leukemic cells involves activation of the apoptotic switch. Molecular studies have revealed that protein-protein interactions between anti-apoptotic and pro-apoptotic proteins are partly responsible for resistance to apoptosis. This finding provided the rationale for identifying compounds such as BH3 mimetics (ABT-737 and ABT-199) that could mimic the BH3 domain of pro-apoptotic proteins of the Bcl-2 family [33]. Proof of this principle demonstrated success in both preclinical and clinical investigations. Similarly, synthetic Smac mimetics reactivate apoptosis in tumor cells by antagonizing IAP family proteins. They act to facilitate apoptosis through one of three mechanisms. Firstly, involves their competitive binding to caspases and antagonizing IAPs. Secondly, involves autoubiquitylation and proteasomal degradation of cIAPs via promotion of E3 ligase activity, leading to RING domain dimerization and concurrent degradation [34], [35]. This mechanism modulates the activity of nuclear factor κB, with consequent stimulation of TNFα, which in turn promotes formation of a receptor-interacting serine-threonine-protein kinase 1 (RIPK1)-dependent caspase-8-activating complex, leading to activation of caspases 3, 7 and 8 [36]. Thirdly, involves activation of the cell's extrinsic apoptotic pathway by autocrine TNFα stimulation or the presence of TNF or TRAIL in the tumor microenvironment. Compared with Smac peptides, small-molecule mimetics have superior affinity for IAPs, cell permeability, *in vivo* stability, and oral activity.

The Smac mimetic, smac066, used in the present study, induces apoptosis as a single agent (Figure 2) by promoting degradation of IAPs in primary CLL cells (Figure 3C-3E). However, Z-VAD-fmk experiments revealed that smac066 is a specific antagonist of IAPs but not Mcl-1, as Z-VAD-fmk reversed caspase-mediated cleavage of Mcl-1 but not IAP proteins (Figure 4B and 4C). There was a strong association between apoptosis, IAP degradation and concurrent caspase activation (Figures 2 and 3).

Full-length XIAP is known to directly inhibit caspases 3, 7, and 9; cIAP1 inhibits caspases 3 and 7; and cIAP2 inhibits caspases 3, 7, and 9. XIAP, cIAP1, and cIAP2 do not inhibit caspases 1, 6 and 8[37, 38]. In our studies, although smac066 is synthesized to bind to BIR3 domains of IAPs, [24] which are required for inhibition of caspase-9, it concurrently induced activation of caspases-8 and 3. It is possible that both activation of caspase-9 or degradation of cIAP1/2 and concurrent activation of caspase-8 [12] converge in a common pathway that ultimately leads to apoptosis by inducing the “executioner” caspases, caspase-3 and −7. Furthermore, active caspase-8 may cleave the pro-apoptotic BH3 only protein Bid to truncated Bid (tBID) which can translocate into the mitochondria, triggering activation of the intrinsic pathway that leads to cytochrome c release and apoptosome formation and ultimately to activation of caspases-9 and 3. Of note, Smac mimetic has been shown to overcome apoptosis resistance in caspase 8-deficient cells by priming TNFα to induce caspase-independent necroptosis or sensitizing apoptosis-proficient cells to TNFα-mediated caspase-dependent apoptosis [39].

A growing body of evidence indicates that there is a fatal attraction between leukemia cells and the “feeder cells” that reside in the compartments of lymph nodes and the bone marrow. These feeder cells, called nurse-like cells or stromal cells, support leukemic cells with nutrients and drug resistance signals. In the present study, two model systems that mimic the bone marrow and lymph node microenvironments respectively, showed enhanced cell survival (Figure 6A-6B). In addition, smac066-induced apoptosis was partially abrogated by stromal co-cultures (Figure 6A-6B). We previously demonstrated that Mcl-1 protein was induced in the presence of stroma, and this induction was partially responsible for enhanced chemoresistance [40]. In this study both XIAP and cIAP2 proteins were significantly induced in co-cultured cells. In the same line, caspase cleavage was diminished. The induction of IAPs with stroma co-cultures was not reversed by smac066 (Figure 6D; n=3; compare lane 3 and 4 for each patient). This finding suggests that a surplus amount of IAPs is induced in the microenvironment, and as a result, smac066 is unable to balance the pro- and anti-survival mechanisms.

Several small-molecule mimetics (monomers or dimers) are in development for treatment of hematological malignancies [17, 41] and solid tumors [42–46]. Birinapant, a bivalent Smac mimetic with high affinity for IAPs, acts through degradation of cIAP1 and caspase-8 activation in acute myeloid leukemia [41]. Birinapant is currently used in the clinic for patients with lymphoma or solid tumors, alone and in combination with other agents [47, 48]. A phase I/II clinical trial of Birinapant as a single agent is launched for elderly patients with AML. LCL161, GDC-0917, HGS1029, and AT-406 are other agents that are currently being evaluated in phase I studies, alone or in combinations, for patients with advanced malignancies. The therapeutic potential and clinical utility of these agents will be understood shortly with the clinical trials that are currently ongoing.

## PATIENTS AND METHODS

### Drugs and chemicals

Smac066 for *in vitro* use was kindly provided by P. Seneci, CISI scrl, University of Milan, Italy. The final concentration of vehicle (DMSO; Sigma-Aldrich (St. Louis, MO)) in untreated and treated samples was 0.1%.

### Patients and healthy donors

This study involved lymphocytes obtained from healthy donors and patients with CLL. CLL or normal PBMCs were isolated from peripheral blood by Ficoll-hypaque gradient method and re-suspended in 10% autologous plasma in RPMI media as described previously [49]. All patients participated in the study had signed written informed consent forms in accordance with the Declaration of Helsinki, and the laboratory protocols were approved by the IRB at the UT MD Anderson Cancer Center. All patients were included in the study or experiments irrespective of treatment status, age or prognosis.

### Marrow Stromal Cell (MSC) co-culture

For CLL-stromal co-culture studies, lymphocytes were incubated without or with confluent layers of human Marrow Stromal Cells (MSC; NKtert; RIKEN cell bank, Tsukuba, Japan) at the ratio of 100 CLL-cells to 1 MSC [49]. The NKTert cell line has been maintained and routinely checked for *Mycoplasma* infection and authenticated by short tandem repeat (STR) analysis at MD Anderson Cancer Center's “characterized cell line core facility”.

### Measurement of cell viability

CLL cell viability was analyzed as described previously by standard methods: annexin V/propidium iodide (PI) binding and 3,3-dihexyloxocarbocyanine iodide (DiOC6) staining, based on the analysis of mitochondrial transmembrane potential and cell membrane permeability to PI [50].

### Bax/Bak wild-type and double-knockout mouse embryo fibroblasts

Exponentially growing Bax/Bak wild-type or double-knockout cell lines (obtained from John C. Reed, Sanford-Burnham Medical Research Institute, San Diego, CA) were used for apoptosis and growth inhibition assays with smac066.

### Nurse-like cells

Nurse-like cell cocultures were established by suspending peripheral blood mononuclear cells (PBMCs) from patients with CLL in complete RPMI 1640 medium with 10% fetal bovine serum and penicillin-streptomycin-glutamine (HyClone) to a concentration of 10^7^ cells/mL (total 2 mL). The cells were monitored, and fresh medium was added as necessary. Cells were incubated for 2 weeks in 24-well plates (Corning Life Sciences) as previously described [51].

### Immunoblot analysis

Cell pellets were lysed in RIPA lysis buffer and the protein content was determined using a DC protein assay kit (Bio-Rad Laboratories). The proteins were run on electrophoresis gels and transferred to nitrocellulose membranes (GE Osmonics Labstore) as described previously [50]. Membranes were blocked for 1 hr in blocking buffer, incubated with primary antibodies against XIAP (BD Biosciences, 610762; as per company, this antibody detects two bands and the lower band is specific for XIAP, as indicated by arrows in the figures); cIAP1 (Abcam, ab2399); cIAP2 (Epitomics, S2700; as per company, this antibody detects two bands and the upper band is specific for cIAP2, as indicated by arrows in the figures); Smac (BD Biosciences, 612246); caspase 3, (Cell Signaling, 9665) caspase 8 (Cell Signaling 9746), and caspase 9 (Cell Signaling, 9502); polyclonal antibody to Mcl-1 or Bcl-xL; and mouse monoclonal antibody to Bcl-2 (Santa Cruz, CA) or to GAPDH (Abcam, Cambridge, MA). The antibody to poly(ADP-ribose) polymerase was from BIOMOL International (Plymouth Meeting, PA). Following washing with PBST, membranes were incubated for 1 hr with infrared dye-labeled secondary antibodies (LI-COR Biosciences), scanned, and visualized using a LI-COR Odyssey infrared imager.

### Immunoprecipitation

Protein–protein interaction between caspases and IAPs was analyzed by immunoprecipitation experiments. Protein G Immunoprecipitation Kit (Sigma-Aldrich) was used according to the manufacturer's instructions.

### Statistical analysis

Linear regression analysis and paired student's t-tests (two tailed) were performed by GraphPad Prism 6 software (GraphPad Software, Inc. San Diego, CA). The statistical analysis for each graph and error bars are denoted in figure legends for the respective figures. Sample size, patient number and prognosis are provided for every experiment included.
